# Feasibility, safety and accuracy of a novel robotic navigation system for cone-beam CT-guided and TACE-assisted radiofrequency ablation of hepatocellular carcinoma, a preliminary study

**DOI:** 10.1186/s12876-026-04956-6

**Published:** 2026-05-23

**Authors:** Zechuan Liu, Tianshi Lyu, Liusong Hu, Siyuan Fan, Sai Zhou, Yuxi Sun, Wenxiu Deng, Li Song, Xiaoqiang Tong, Yinghua Zou, Jian Wang

**Affiliations:** Department of Interventional and Vascular Surgery, Peking University First Hospital, Beijing, China

**Keywords:** Hepatocellular carcinoma, Robotic navigation, CBCT guidance, TACE assistance, Radiofrequency ablation

## Abstract

**Purpose:**

To evaluate the technical feasibility, targeting accuracy and short-term safety of a novel integrated system of the robot-navigated, cone-beam CT (CBCT)-guided and transarterial chemoembolization (TACE)-assisted radiofrequency ablation (RFA) for hepatocellular carcinoma (HCC) in high-risk locations.

**Materials and methods:**

This retrospective, single-center study enrolled 11 participants with HCC lesions in high-risk locations. All participants underwent TACE followed by RFA navigated by a novel robotic system. Trajectory planning was based on respiratory-gated CBCT images and the subsequent needle insertion was guided by real-time fluoroscopy. The evaluated outcomes included the first placement accuracy, the adequate placement accuracy, targeting time, repositioning times, radiation exposure dose, complications and the 1-month complete ablation rate.

**Results:**

The cohort included 10 males and 1 female. Technical success was achieved in 100% (11/11) of cases. The median tumor diameter was 16.20 mm and the mean tumor depth was 79.37 mm. Seven tumors were located in the subdiaphragmatic position, three tumors were situated adjacent to major vascular structures and one tumor was located adjacent to extrahepatic organs. The median Euclidean error and angle error of the first placement were 6.2 mm and 3.0 degrees, respectively. The median Euclidean error and angle error of the adequate placement was 2.2 mm and 1.3 degrees. The median targeting time was 6.70 min (range, 1.55–29.52 min) and the median number of repositioning adjustments was 2. The mean cumulative air kerma was 216.34 mGy. The 1-month complete ablation rate was 81.8% (9/11) following adjunctive ablation in 2 patients. No major complications were reported.

**Conclusion:**

The integrated platform of robotic-navigated, real-time CBCT-guided, and TACE-assisted RFA is technically feasible and demonstrates short-term safety. In this cohort, it achieves preliminary targeting accuracy for the ablation of HCC in high-risk locations.

## Introduction

Hepatocellular carcinoma (HCC) represents a major global health burden, ranking as the sixth most common cancer and the third leading cause of cancer-related mortality worldwide [1]. Surgical resection and liver transplantation are considered curative treatments, but a large proportion of patients are ineligible due to advanced disease, poor liver function, or donor scarcity. Therefore, locoregional therapies have become indispensable. Percutaneous thermal ablation, particularly radiofrequency ablation (RFA), has emerged as a first-line, curative treatment for early-stage HCC [2, 3].

The main drawback of percutaneous liver tumor ablation is local recurrence of HCC, with reported recurrence rates ranging from 5.0% to 30% [4–6]. A critical factor for local recurrences is the failure to achieve an adequate ablation margin surrounding the tumor [7–9]. This challenge is multifactorial. Firstly, it can be caused by the inaccurate placement of the ablation antenna. Currently, antenna placement is mostly performed manually under CT or ultrasound guidance. However, achieving the intended trajectory remains challenging, particularly for lesions requiring complex or out-of-plane trajectories. Secondly, the application of RFA is frequently constrained by tumor location. HCCs situated in “high-risk” locations, such as the subdiaphragmatic or subcapsular regions, are often poorly visualized or entirely obscured on conventional ultrasonography. This poor visibility hinders the precise targeting required for complete ablation.

To overcome these limitations, advanced image guidance and robotic navigation technologies have been introduced [10]. They generally enhance positioning accuracy, reduce procedural time, and/or minimize operator radiation exposure [11–13]. However, the application of robotic navigation remains constrained in liver tumor ablation. The primary challenge is significant respiratory motion, which creates a critical mismatch between the static plan and the dynamic target [14]. Despite the robotic navigation system integrates sophisticated respiratory monitoring and gating technology, residual motion can still severely compromise the high-precision hepatic interventions.

Based on these issues, we proposed a novel integrated strategy to enhance the accuracy and safety of robotic-navigated ablation for HCC, which utilizes the integration of conventional transarterial chemoembolization (c-TACE) and cone-beam CT (CBCT) to achieve respiratory gating and real-time guidance during robotic-assisted puncture. This integrated strategy is expected to enhance the safety and accuracy of robot-assisted percutaneous interventions in abdominal organs with high respiratory mobility by implementing real-time respiratory gating. This study aims to evaluate the clinical feasibility, preliminary accuracy, and short-term safety of the robot-navigated, CBCT-guided and TACE-assisted RFA for HCC in high-risk locations.

## Materials and methods

### Study design and patient population

**Fig. 1:**
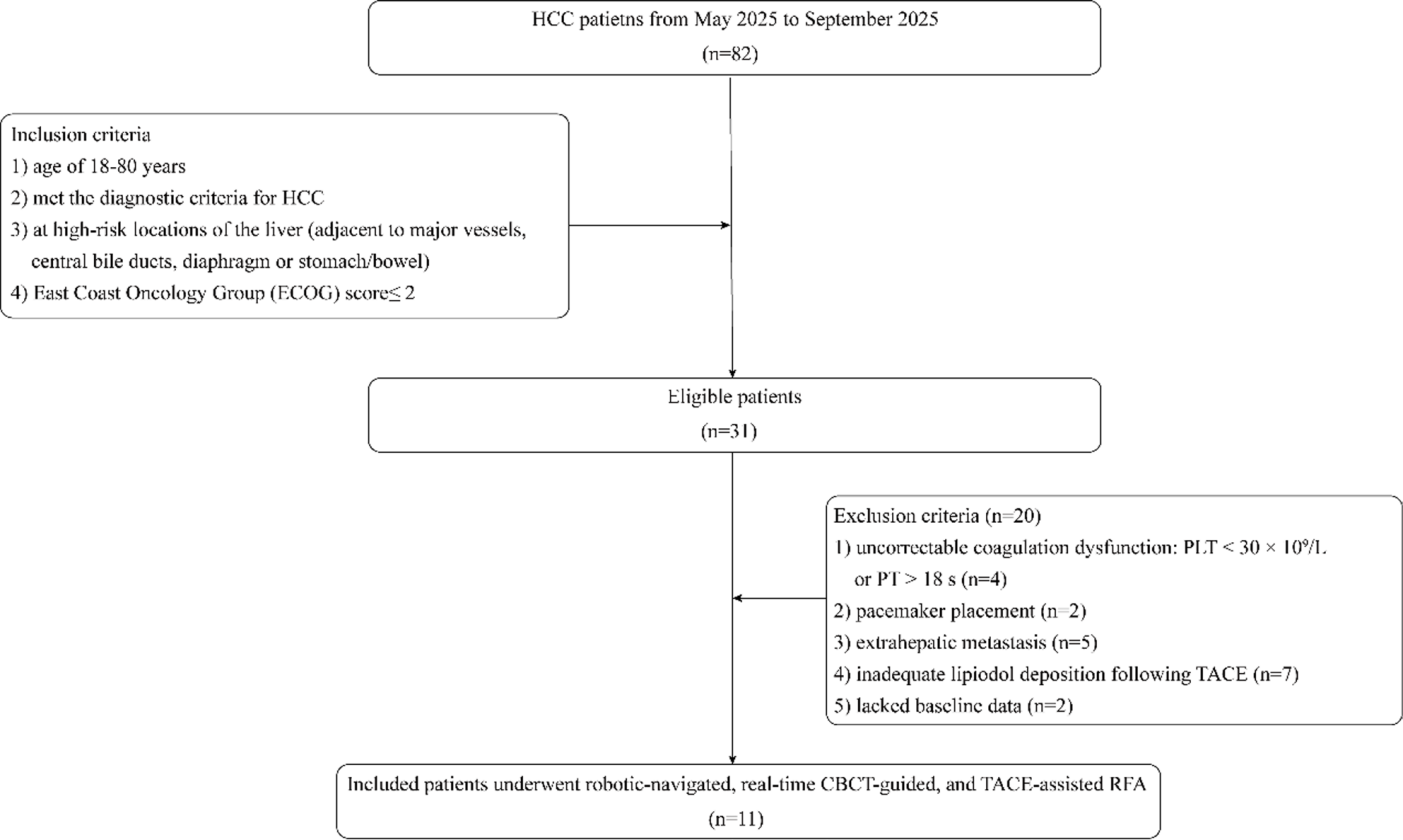
Patient flow diagram. HCC, hepatocellular carcinoma; PLT, platelet; PT, prothrombin time; TACE, transarterial chemoembolization; CBCT, cone-beam CT; RFA, radiofrequency ablation

This retrospective, single-center study was conducted in accordance with the Declaration of Helsinki of the World Medical Association and received approval from the Institutional Review Board. We enrolled the patients between May 2025 and September 2025. The inclusion criteria were as follows: (1) age of 18–80 years; (2) met the diagnostic criteria for HCC; (3) HCC at high-risk locations of the liver; (4) Eastern Cooperative Oncology Group (ECOG) score ≤ 2. The exclusion criteria were as follows: (1) uncorrectable coagulation dysfunction: PLT < 30 × 10^9^/L or PT > 18 s; (2) pacemaker placement; (3) extrahepatic metastasis; (4) inadequate lipiodol deposition following TACE; (5) lack of baseline data. Eleven patients were included in this study based on the inclusion and exclusion criteria (Fig. 1). High-risk sites were defined as HCC closely adjacent to critical structures, including major vessels (porta veins, hepatic veins), central bile ducts, diaphragm, or stomach/bowel [15].

### Robotic navigation system

**Fig. 2:**
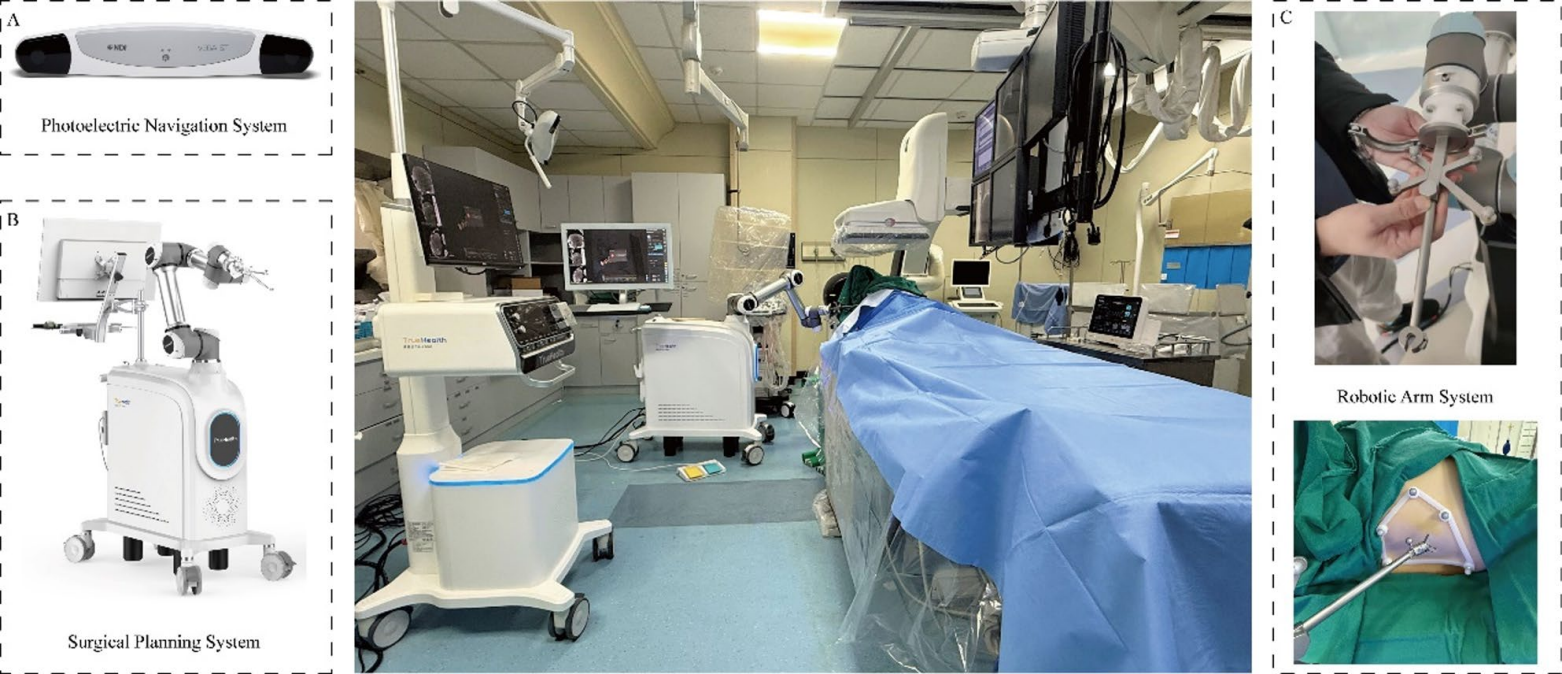
Robotic-assisted navigation system. (**A**) The photoelectric navigation system for real-time tracking. (**B**) The surgical planning system for 3D reconstruction and path planning. (**C**) The robotic arm system for precise positioning and puncture

The robotic navigation positioning system (Guangdong True Health Medical Technology Development Co., Ltd. Hengqin, China) was used for trajectory planning and percutaneous puncture in this study. The surgical robotic arm features six degrees of freedom, with a system accuracy of ≤ 0.8 mm, a position accuracy error of ≤ 0.5 mm, and a position repeatability error of ≤ 0.2 mm. The robotic system consists of three parts (Fig. 2): (1) a photoelectric navigation system for real-time tracking; (2) a software planning system for 3D reconstruction and path planning; (3) a robotic arm for precise positioning and puncture.

The workflow of the robotic navigation system are as follows: (1) Preoperative CBCT images are acquired and subsequently imported into the software planning system; (2) the position information of the patient obtained through the photoelectric navigation system is registered with the 3D model in real time; (3) the physician plans the optimal placement trajectory in the software planning system; (4) the robotic arm positioning system registers the planned puncture trajectory (including the needle insertion point, angle, and depth) in the operational space; (5) the physician inserts the ablation needle under the guidance of the robotic arm and real-time fluoroscopy.

### Procedure

#### c-TACE

Under local anesthesia, standard conventional TACE (c-TACE) was performed via a femoral approach. Following super-selective catheterization, the tumor-feeding arteries were embolized using the emulsion of lipiodol and epirubicin. Supplementary embolization was then performed using particulate embolic agents. CBCT was utilized to confirm the lipiodol deposition within the tumor, which subsequently served as the fiducial marker, rendering the HCC clearly visible under fluoroscopy and CBCT.

#### Robotic-navigated and CBCT-guided RFA

Within one week following TACE, the patient underwent robotic-navigated and CBCT-guided RFA. A supine or lateral position was selected based on the intrahepatic lesion’s location.

1. Image acquisition and trajectory planning. A respiratory-gated CBCT scan (e.g., acquired at end-inspiration) was performed. The CBCT was performed using a flat-panel digital angiography system (Innova 540, GE Healthcare). The acquisition utilized a 6-second protocol with a rotation speed of 40°/s, followed by thin-slice reconstruction at 0.625 mm. The DICOM data were transferred to the robotic workstation. The 3D model was reconstructed, and the operator planned the optimal puncture trajectory.
2. Robotic arm navigation and placement. The robotic arm automatically adjusted the needle guide to align with the planned trajectory.
3. Real-time respiratory guidance and puncture. Crucially, both breath gating and subsequent needle insertion were performed under real-time X-ray fluoroscopy. Initially, the operator instructed the patient to regulate their breathing amplitude to precisely match the respiratory state captured during the CBCT planning. Subsequently, the operator inserted the RFA electrode to the predetermined depth under real-time fluoroscopic visualization.
4. Ablation. Once the needle tip was confirmed by the CBCT, RFA was performed according to the manufacturer's protocol. If necessary, the needle was then repositioned for further ablation.

### Accuracy assessment and follow up

**Fig. 3:**
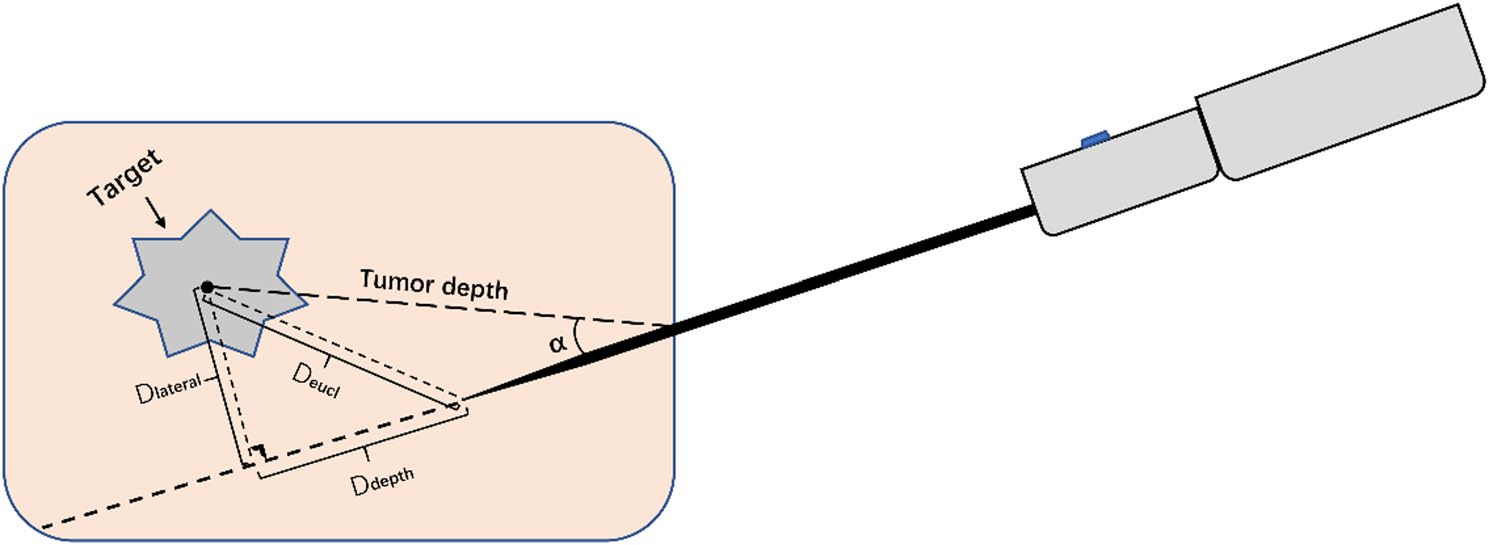
Schematic of accuracy measures. Angle error (α) is the angular deviation between the actual needle trajectory and the planned trajectory. Tumor depth is the distance between the skin entry point and the target point. D_eucl_ is the Euclidean distance between the actual needle tip position and the target point. D_depth_ is the distance of D_eucl_ projected along the actual needle trajectory. D_lateral_ is the distance of D_eucl_ projected perpendicular to the actual needle trajectory

CBCT scans were performed following the first placement and adequate placement of the needle. Adequate placement was defined as positioning the ablation electrode within or immediately adjacent to the target tumor, ensuring sufficient ablation zone. If multi-point ablation was necessary, the repositioning of the ablation antenna was performed manually. Procedural data were collected retrospectively and uploaded to the surgical verification software. The deviations between the planned and actual placement were measured, as illustrated in Fig. 3. Tumor depth was determined as the distance between the skin entry point and the target point. The alpha angle (α) was defined as the angular deviation between the actual needle trajectory and the planned trajectory. The D_eucl_ was defined as the Euclidean distance between the actual needle tip position and the target point. D_depth_ and D_lateral_ were respectively defined as the distances of D_eucl_ projected along the actual needle trajectory and perpendicular to it. Imaging outcomes were evaluated by two independent radiologists blinded to the procedural details. Any disagreements between the two reviewers were decided by a third independent expert to ensure objective assessment. Technical success was defined as the accurate placement of the ablation electrode within the HCC and the successful completion of the robotic-navigated RFA. Complete ablation was defined as the absence of arterial contrast enhancement within the HCC according to mRECIST criteria, as assessed by contrast-enhanced CT or MRI at one-month post-RFA.

### Statistical analysis

Categorical variables were presented as frequencies and percentages. Continuous variables were presented using standard descriptive statistics, including means, standard deviations, median, minimum and maximum values. Proportions and their corresponding 95% confidence intervals (CIs) were calculated using the Clopper-Pearson exact method due to the small sample size. Statistical analysis and description were conducted using Stata/SE software (version 15.0) and R statistical software (version 4.1.3).

## Results

### Patient and tumor characteristics

Between May 2025 and September 2025, eleven participants were included in our study, who underwent robot-navigated, CBCT-guided and TACE-assisted RFA in Peking University First Hospital. Seven patients were excluded because of poor lipiodol uptake post-TACE, making the lesions indistinguishable under fluoroscopy. Table 1 shows the participant characteristics. All patients had a Child-Pugh score of 5 (Class A). Of these, seven patients were classified as ALBI Grade 1, and four patients were classified as ALBI Grade 2. Table 2 provides a summary of the tumor characteristics. Among all eleven tumors, the median diameter was 16.20 mm (range, 10.20–96.50 mm) and the mean tumor depth was 79.37 mm. All lesions were situated in high-risk locations: seven tumors (63.6%) were subdiaphragmatic, three tumors (27.3%) were adjacent to major vascular structures, and one tumor (9.1%) was adjacent to the kidney. According to clinical indications, nine tumors underwent curative ablation and two tumors underwent adjunctive ablation.

**Table 1:** Participant characteristics

Characteristics	Value
Sex, n (%)	
Male	10 (90.9)
Female	1 (9.1)
Age, years*	60.3 ± 8.8
Etiology of cirrhosis, *n* (%)	
HBV	8 (72.7)
HCV	3 (27.3)
Child-Pugh score	5.0
ALBI, *n* (%)	
Grade 1	7 (63.6)
Grade 2	4 (36.4)
ALB (g/L)*	39.05 ± 3.39
TBil (umol/L)*	12.19 ± 4.76
PLT (10^9^/L)*	130.64 ± 68.84
PT (s)*	11.39 ± 0.85

* Data are means ± standard deviations

**Table 2:** Tumor characteristics

Characteristics	Value
No. of tumors	11
Tumor location, *n* (%)	
S4	2 (18.2)
S6	1 (9.1)
S7	3 (27.3)
S8	5 (45.4)
BCLC grade, *n* (%)	
A	4 (36.4)
B	7 (63.6)
Tumor diameter, (mm)	16.20 (10.20–96.50)
Tumor depth, (mm)*	79.37 ± 15.22
Adjacent high-risk structures	
Diaphragm	7 (63.6)
Major vascular structures	3 (27.3)
Extrahepatic organs	1 (9.1)
AFP (ng/mL)	4.87 (1.93–959.00)
PIVKA (mAU/mL)	88.70 (22.04-2769.7)
Treatment target, *n* (%)	
Curative ablation	9 (81.8)
Adjunctive ablation**	2 (18.2)

* Data are means ± standard deviations

** Ablation for the residual tumor portions following past local therapy of the large HCC

### Technical outcomes of robotic procedures

**Fig. 4:**
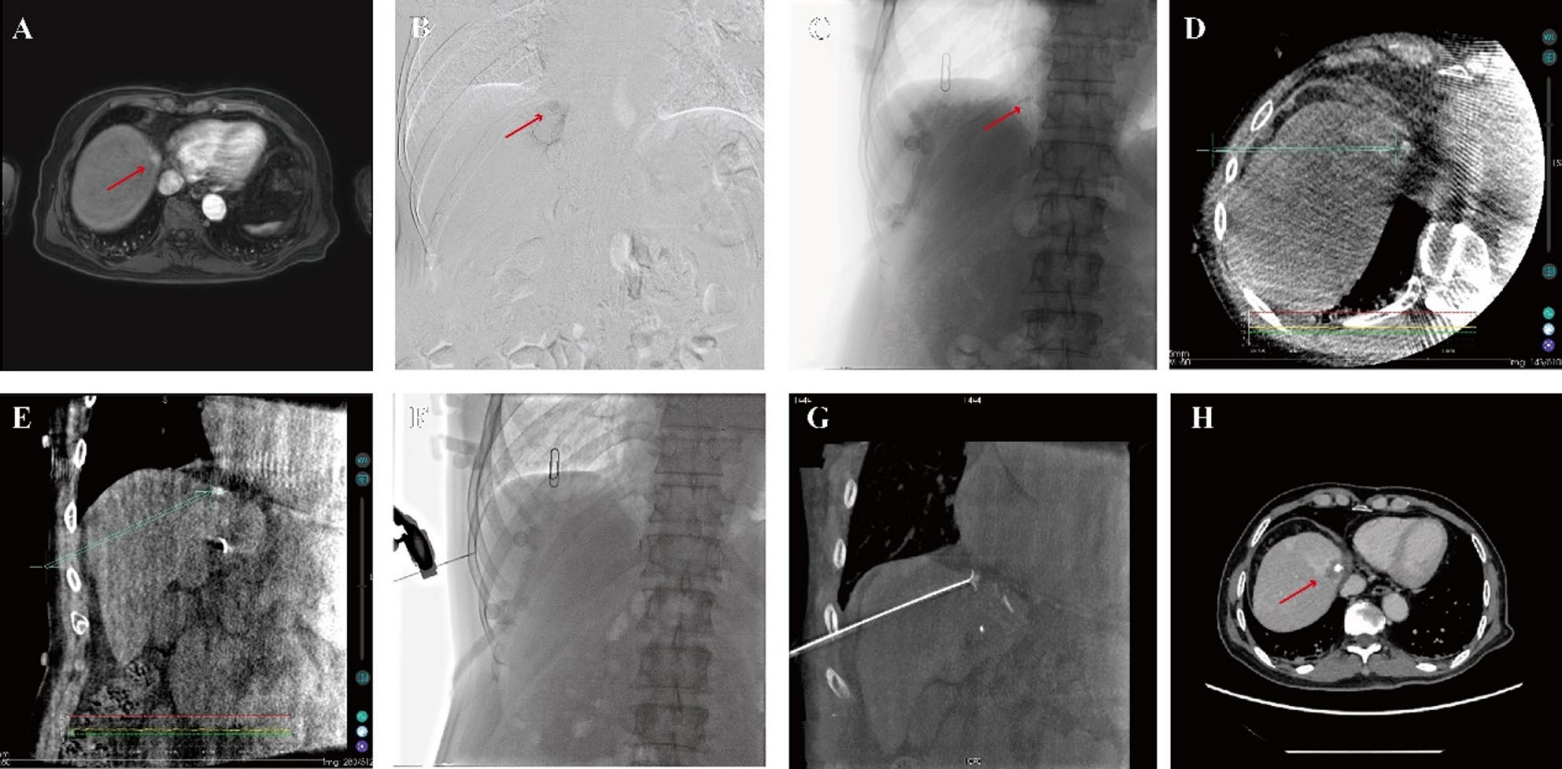
A case of robotic-navigated, real-time CBCT-guided, and TACE-assisted RFA for recurrent HCC following surgical resection in a 79-year-old male. (**A**) Contrast-enhanced MRI showed the recurrent HCC lesion adjacent to the surgical margin, situated in the subphrenic region and in close proximity to the pericardium. (**B**) Angiography revealed significant tumor staining (arrow). (**C**) CBCT demonstrated lipiodol deposition within the lesion (arrow). **D**, **E**. Trajectory planning was performed on preoperative CBCT images using the robotic navigation system. **F**. Needle insertion was performed under the guidance of TACE-assisted respiratory gating. **G**. The needle accurately targeted the lesion according to the preoperative planning trajectory. **H**. One-month follow-up CT demonstrated complete ablation of the recurrent HCC

**Fig. 5:**
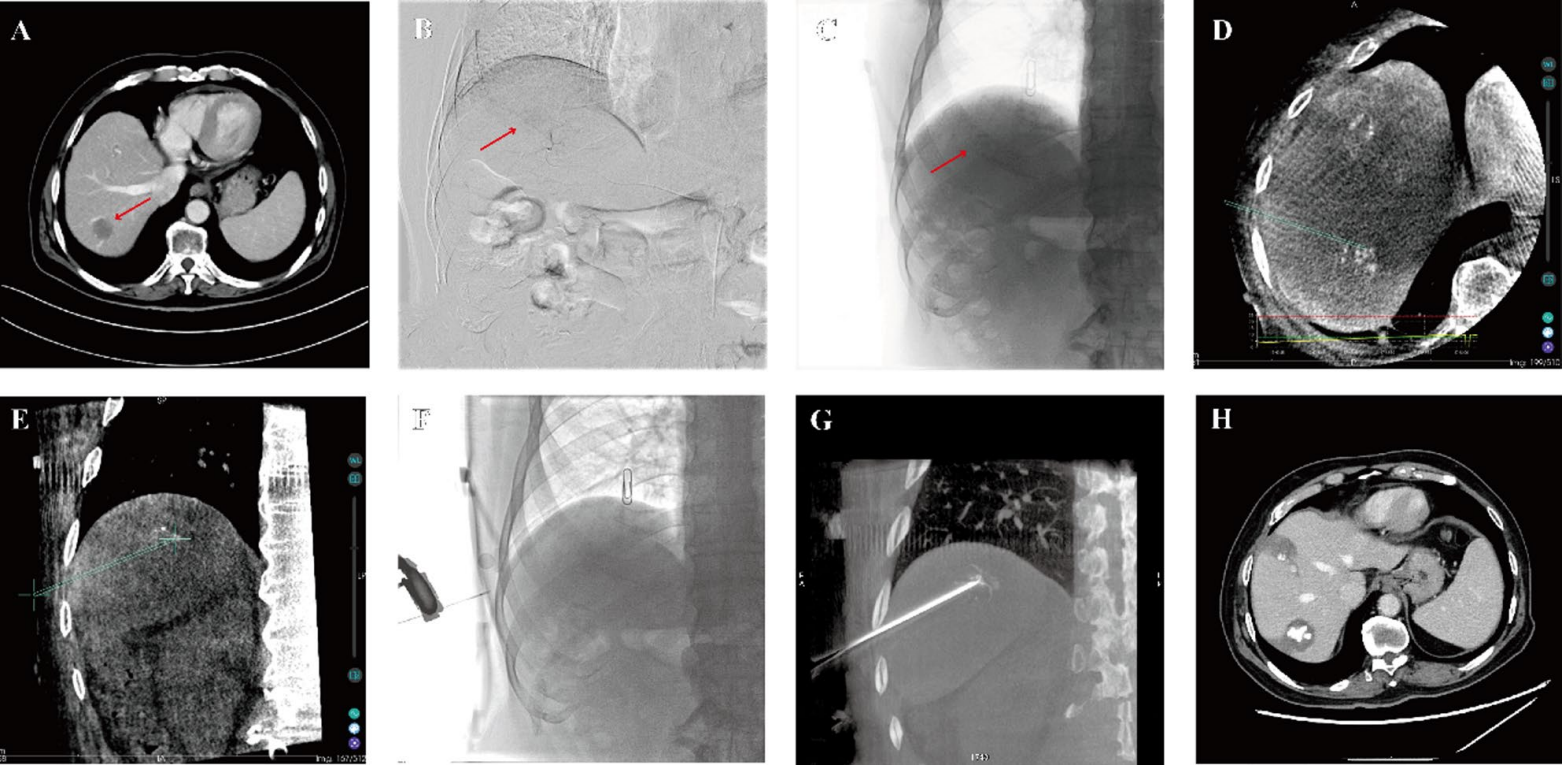
A case of robotic-navigated, real-time CBCT-guided, and TACE-assisted RFA for perivascular HCC near the right hepatic vein in a 64-year-old male. (**A**) Contrast-enhanced CT showed an HCC adjacent to the right hepatic vein (S7). (**B**) Angiography revealed significant tumor staining in S7 (arrow). (**C**) CBCT demonstrated lipiodol deposition within the lesion (arrow). **D**, **E**. Trajectory planning was performed on preoperative CBCT images using the robotic navigation system. **F**. Needle insertion was performed under the guidance of TACE-assisted respiratory gating. **G**. The needle accurately targeted the lesion according to the preoperative planning trajectory. **H**. One-month follow-up CT demonstrated complete ablation of the perivascular HCC

**Fig. 6:**
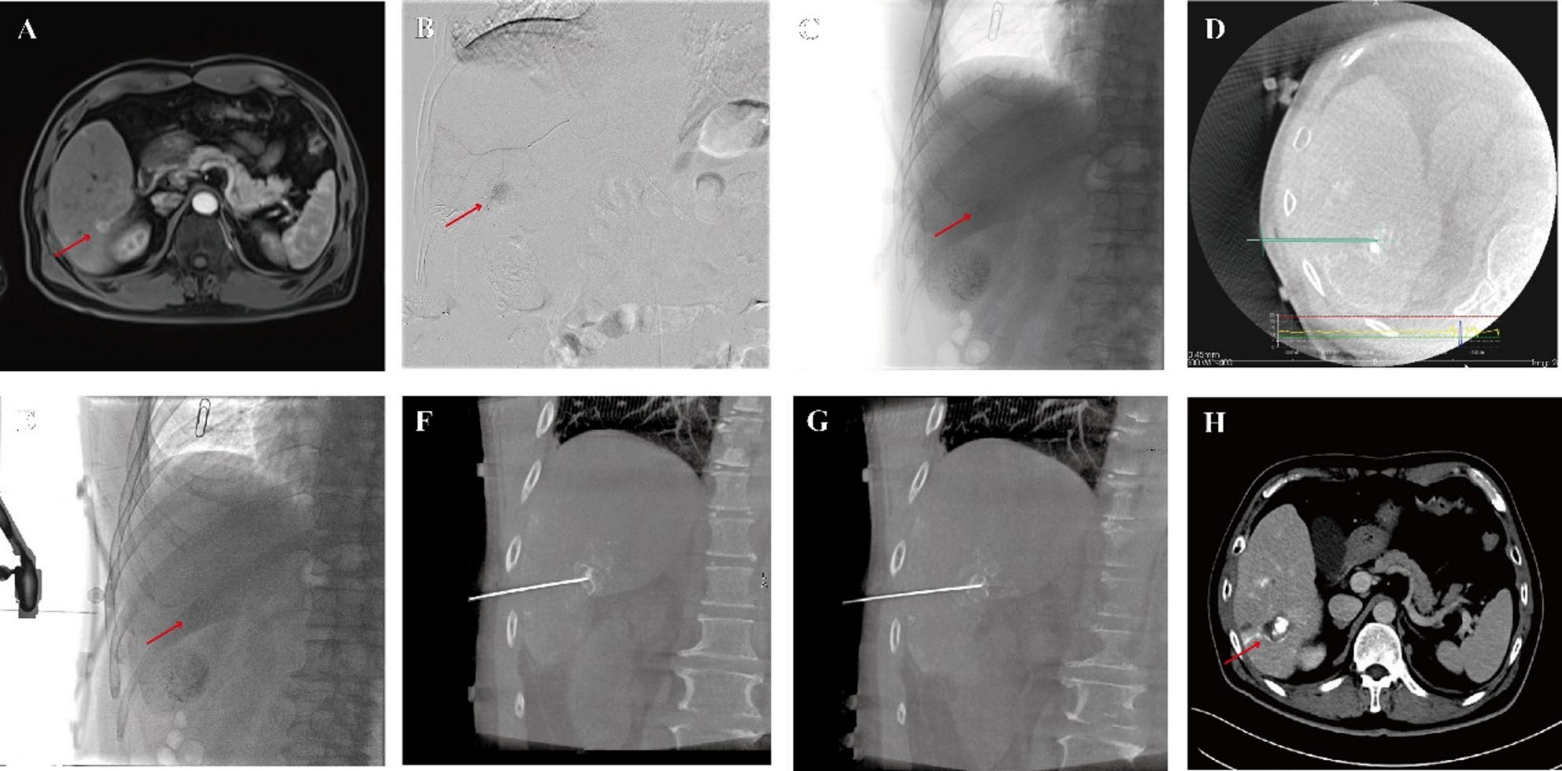
A case of robotic-navigated, real-time CBCT-guided, and TACE-assisted RFA for the HCC adjacent to the right kidney in a 54-year-old male. (**A**) Contrast-enhanced MRI showed an HCC adjacent to the right kidney (S6). (**B**) Angiography revealed significant tumor staining in S6 (arrow). (**C**) CBCT demonstrated lipiodol deposition within the lesion (arrow). (**D**) Trajectory planning was performed on preoperative CBCT images using the robotic navigation system. (**E**) Needle insertion was performed under the guidance of TACE-assisted respiratory gating. **F**, **G**. The needle accurately targeted the lesion and two-site radiofrequency ablation was performed. **H**. One-month follow-up CT demonstrated complete ablation of the HCC

**Fig. 7:**
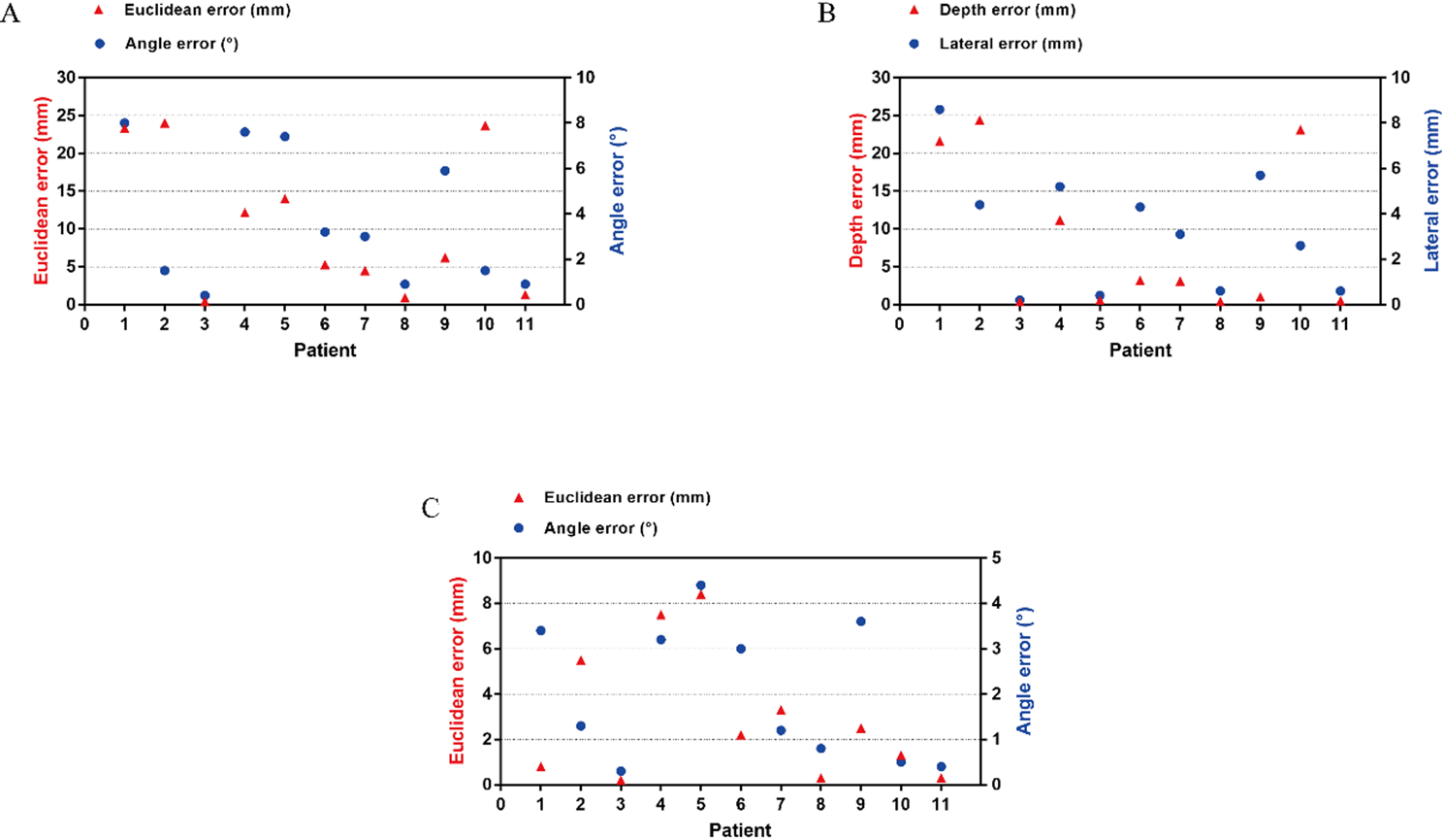
Deviations between the planned and placed puncture. (**A**) The angle error (mm) and Euclidean error (°) of the first placement. (**B**) The lateral error (mm) and depth error (mm) of the first placement. (**C**) The angle error (°) and Euclidean error (mm) of the adequate placement

A series of cases illustrating robotic-navigated, real-time CBCT-guided, and TACE-assisted RFA for HCC in high-risk locations was presented in Figs. 4, 5 and 6. Technical success was achieved in 100% (11/11, 95% CI: 71.5%-100.0%) of cases. Technical outcomes for the robotic procedures are summarized in Table 3. The median number of needle repositions was two (range, one to four). The median targeting time to achieve the first placement was 6.70 min (range, 1.55–29.52 min), and the mean total procedure time was 59.93 min. A median of three CBCT scans were performed to ensure adequate needle placement. For the first placement, the median Euclidean error was 6.2 mm (range, 0.4–24.0 mm) and the median Angle error was 3.0 degrees (range, 0.4-8.0 degrees) (Fig. 7A). The median lateral error was 3.1 mm (range, 0.2–8.6 mm) and the median depth error was 3.1 mm (range, 0.4–24.4 mm) (Fig. 7B). Following needle repositioning, the adequate placement accuracy was further enhanced, specifically, the median Euclidean error decreased to 2.2 mm, and the median angle error was reduced to 1.3 degrees (Fig. 7C). The mean cumulative air kerma exposure was 216.34 mGy.

**Table 3:** Technical outcomes of robotic procedures

Characteristics	Value
Technical success, *n* (%)	11 (100)
First placement accuracy	
Euclidean error (mm)	6.2 (0.4–24.0)
Lateral error (mm)	3.1 (0.2–8.6)
Depth error (mm)	3.1 (0.4–24.4)
Angle error (degrees)	3.0 (0.4-8.0)
Adequate placement accuracy	
Euclidean error (mm)	2.2 (0.2–8.4)
Angle error (degrees)	1.3 (0.3–4.4)
Targeting time (min)*	6.70 (1.55–29.52)
Total procedure time (min)**	59.93 ± 18.41
No. of needle repositions	2 (1–4)
No. of CBCT scans	3 (1–6)
Total fluoroscopy time (min)**	2.53 (1.33–9.40)
Cumulative air kerma (mGy) **	216.34 ± 79.53
Dose area product (Gy. cm^2^) **	63.27 ± 15.91
Follow-up after 1 month, *n* (%)	
Complete ablation	9 (81.8)
Incomplete ablation	2 (18.2)

* Time to achieve the first placement

** Parameters refer to the robotic-navigated RFA session and exclude TACE procedure

### Safety and clinical outcomes

No major complications were observed during the procedures (0/11, 95% CI: 0.0%-28.5%). At the 1-month follow-up, complete ablation was achieved in nine HCCs (81.8%, 95% CI: 48.2%-97.7%). The remaining two HCCs (18.2%) were classified as incomplete ablation. Both patients had large-burden HCC (diameter > 10 cm) and a history of multiple TACE and ablation sessions. The primary target of the current therapy was adjunctive ablation of the partial residual tumor.

## Discussion

This preliminary study demonstrates that the integrated platform combining robotic navigation, real-time CBCT guidance and TACE-assistance is technically feasible, safe, and accurate for RFA of HCC in high-risk locations. Firstly, we performed conventional transarterial Chemoembolization (c-TACE) for HCCs. Subsequently, we used fluoroscopy and cone-beam CT (CBCT) during the robotic-assisted puncture. This combined strategy enabled the operator actively monitor the patient’s breathing and the target HCC in real-time, thereby ensuring the respiratory depth precisely match the planning CBCT images to achieve accurate puncture and ablation of the HCC. Our approach achieved 100% technical success, with no major complications. The first placement showed a Euclidean error of 6.2 mm and an angular error of 3.0 degrees.

The efficacy and safety of thermal ablation for HCC lesions situated in high-risk anatomical locations are limited. Ablation in high-risk sites was typically performed by reducing the power, range, or duration of the procedure to mitigate the thermal damage to surrounding tissue, such as diaphragmatic injury, pleural effusion, or organ perforation [16]. However, this approach is often associated with inadequate ablation, which can lead to suboptimal outcomes [17]. Consequently, the anatomical constraints and associated risks in these challenging settings underscore an urgent clinical need for advanced, high-precision guidance and navigation systems.

Robotic navigation systems for percutaneous interventions have expanded rapidly. Preclinical studies in animal models demonstrated that CT-guided robotic needle insertion achieved an accuracy comparable to that of manual insertion, while the number of needle insertions, confirmatory scans, and radiation exposure reduced significantly [18, 19]. Previous studies also reported that robot-assisted puncture exhibited significantly higher success rates and accuracy compared to manual puncture, while also markedly decreasing the cumulative radiation dose [11]. Recently, Liang et al. reported an RCT involving 100 patients which demonstrated that robotic navigation system achieved a higher first-placement success rate and substantially decreased localization time, number of CT scans, and radiation exposure for isolated lung nodule localization [20]. The evidence confirms the high accuracy and favorable safety profile of the robotic navigation platform.

However, the application of the robotic navigation system remains limited in the field of liver tumor ablation, while respiratory motion is the primary hurdle [14, 21]. The diaphragm, as the primary muscle of respiration, induces stereotactic abdominal motion that can alter the spatial location of the liver tumor [22]. Therefore, the planned trajectory and actual puncture path of the robotic navigation system must be coordinated with the patient’s respiratory control. Although several strategies have been established to mitigate respiratory motion, these methods remains cumbersome and does not sufficiently reduce respiratory errors [14, 23]. The efficacy and safety of the robotic navigation system for liver tumors was preliminary studied in previous studies [24, 25]. Abdullah et al. demonstrated a 100% puncture success rate with the robotic-assisted thermal ablation for liver tumors, and no robotic-related complications were reported [26]. Heerink et al. reported an RCT which demonstrated that the robotic group required significantly fewer needle adjustments compared to the freehand group (0 vs. 1), and exhibited a smaller lateral targeting error (5.9 mm ± 2.9 vs. 10.1 mm ± 4.0) [27]. To effectively control the respiratory motion, both of these studies were performed under general anesthesia, and the puncture procedure was conducted during an end-expiratory breath-hold. This specific state was achieved by disconnecting the airway from the ventilator, which was cumbersome and unsafe. Compared with the studies by Heerink and Abdullah, all puncture and ablation procedures in this study were performed under local anesthesia plus intravenous analgesia.

Several critical limitations hinder the widespread clinical adoption of robotic-navigated ablation for liver tumors. Firstly, existing studies predominantly rely on general anesthesia to strictly control respiratory phases, while liver thermal ablation is usually performed under local anesthesia in most medical centers [11, 27, 28]. This reliance not only escalates the anesthetic risk but also increases procedural complexity and the associated economic burden. Secondly, even when mechanical ventilation and induced apnea are employed to manage respiratory motion, the puncture is still conducted in a “semi-blind” state, which is invisible of the whole insertion process. Therefore, there is a lack of real-time intraprocedural visualization to monitor the dynamic spatial relationships between the needle tip and the target tumor. Our integrated strategy directly addresses this challenge. By performing c-TACE first, we make the tumor densely opacified and clearly visible on fluoroscopy and CBCT. During the robotic-guided puncture, the operator uses live fluoroscopy to visually match the position of the opacified HCC and diaphragm to the planning CBCT. This “image-guided breath-hold” strategy provides the real-time verification to ensure the patient’s respiratory phase precisely aligns with the preoperative plan, thereby enabling accurate puncture and ablation.

This study has several limitations. Firstly, this is a preliminary, single-arm retrospective study with a small sample size, the results require validation through larger-scale, multi-center RCT. Secondly, this integrated strategy requires super-selective TACE to ensure dense lipiodol deposition within the HCC. Thirdly, compared with conventional CT- or ultrasound-guided RFA, the workflow for robot-assisted RFA is relatively complex. Consequently, this technique was exclusively reserved for the HCCs located in high-risk areas in the present study.

In conclusion, by integrating robotic navigation with TACE-assisted visualization, the study overcomes the limitations of respiratory gating in conventional robotic-guided procedures. Our findings preliminarily demonstrate that the integrated platform of robotic-navigated, real-time CBCT-guided and TACE-assisted RFA is a technically feasible, safe, and accurate method for treating HCCs in high-risk locations.

## Data Availability

The datasets used and analyzed during the current study are available from the corresponding author on reasonable request.
